# Operational Implications and Risk Assessment of COVID-19 in Dental Practices

**DOI:** 10.3390/ijerph182212244

**Published:** 2021-11-22

**Authors:** Saba Wajeeh, Abhishek Lal, Naseer Ahmed, Md. Ibrahim Khalil, Afsheen Maqsood, Akram Mojidea M Alshammari, Abdulelah Zaid Alshammari, Meshari Musallam Mohammed Alsharari, Abdulelah Hamdan Alrushaydan, Abdulaziz Fandi Alruwaili, Mohammad Khursheed Alam

**Affiliations:** 1 Quality Assurance Department, Altamash Institute of Dental Medicine, Karachi 75500, Pakistan; 2 Department of Prosthodontics, Altamash Institute of Dental Medicine, Karachi 75500, Pakistan; abhishekdarshan@yahoo.com (A.L.); naprosthodontist@gmail.com (N.A.); 3 Departamento de Odontologia, Clínica Internacional CIRO, University De La Salle, Av. Benjamín Franklin 45, Colonia Condesa, Cuauhtémoc, Ciudad de México 06140, Mexico; dr.md.ibrahimkhalil@gmail.com; 4 Department of Oral Pathology, Bahria University Medical and Dental College, Karachi 75530, Pakistan; afsheenmaqsood@gmail.com; 5 Department of Preventive Dental Science, College of Dentistry, Jouf University, Sakaka 72345, Saudi Arabia; dr.akram.alshammari@gmail.com (A.M.M.A.); abdulelah.zaid.alshammari@gmail.com (A.Z.A.); mashary543@gmail.com (M.M.M.A.); abdalelah.hamdan.alrshedan@jodent.org (A.H.A.); abdulaziz0101234@gmail.com (A.F.A.)

**Keywords:** COVID-19 pandemic, dental clinics, dentists, patients, risk assessment

## Abstract

The unprecedented situation of the coronavirus pandemic has impacted the entire world, with dental practice being significantly affected. In this study, we aim to evaluate the operational implications and risk assessment of the coronavirus in dental practice. This observational study comprised the electronic distribution of two surveys, one to patients and the second to dental professionals. The first questionnaire consisted of demographics along with 15 closed-ended questions. The second questionnaire consisted of demographics along with 43 questions from eight domains: financial impact, psychological impact, patient satisfaction, hygiene, patient management, COVID-19 lockdown, perspective, and practicing dentistry after the COVID-19 pandemic. The statistical analysis was performed using SPSS-25. A linear regression test was applied to assess the effect of the dependent variable (patient’s satisfaction with the dental practice) on independent variables (age, gender, education). The ANOVA test was applied to assess the effect of the independent variables (financial impact, psychological impact, patient satisfaction, hygiene, patient management, lockdown, perspective, and post-COVID-19) on the dependent variables (age, gender, education, experience of dentists, qualification of dentists). A total of 711 patients and dental professionals participated in this study, with a response rate of 88.87%. Approximately 67.9% of the patients felt comfortable in the dental clinic, with 74.5% being satisfied with the dental clinic’s services. The majority (77.4%) of the dentists were psychologically affected. Many of the participants chose to use masks, gowns, respiratory equipment, and face shields for protection. Teledentistry was preferred by the majority of dentists in non-emergency cases. Many of the dentists chose alternative procedures to minimize the generation of aerosols. The majority of the dentists suggested changes in the dynamics of dentistry in the post-COVID era, such as the suggestion that the management of the finances of a dental practice along with infection control protocols should be practiced more optimally. Patients and dental professionals were well aware of the necessary precautionary measures required to combat the coronavirus, as well as the implications of different operational measures along with performing risk assessment, keeping in mind the changing dynamics of dentistry.

## 1. Introduction

In Wuhan, China, in December 2019, the reported of the novel coronavirus disease 2019 (COVID-19) began, which has now spread globally and affected every aspect of human life [1]. Recently, to tackle the virus outbreak, a vaccination drive has been started globally in which different manufacturers produce vaccines, as, previously, vaccines have been proven to be effective against different infectious diseases [2].

Coronavirus is known to spread quickly throughout its surroundings, mainly by respiratory airborne droplets when an infected individual coughs and sneezes [3]. A person infected with coronavirus primarily presents with fever, dry cough, sore throat, myalgia, and loss of smell and taste [4]. Coronavirus has been known to mutate, with different strains that are present globally, affecting individuals more than once at times, which affects the efficacy of vaccines [5].

Due to the current pandemic situation, dental practices have been affected to a greater extent since the doctors, as well as the patients, are directly at risk of contracting the virus. Most dental procedures, such as root canal treatment, fillings, and extractions, where dental suction has to be used, generate aerosols, which are the main risk factors for the transmission of COVID-19 [6]. This then necessitates precautionary measures for the protection of patients as well as doctors. Within the dental practice, the use of a face mask, checking of temperature, and proper social distancing, along with hand sanitization, are the most important modalities to decrease the risk of contracting the virus [7]. The use of all these precautionary measures is of vital importance in ensuring a comfortable environment for the patients.

According to the Center for Disease Control and Prevention (CDC), dentists, along with their assistants, are required to wear personal protective equipment (PPE) since they are in direct contact with the patients [8]. Since the pandemic began, dental practices have been affected, with many changes being introduced to manage the practicing of dentistry under these new circumstances. There has been widespread fear of contracting the coronavirus, especially amongst dentists, which generates anxiety and stress, thereby having a great psychological impact. Since dental practices pose a great risk of contracting the virus, it has been advised to treat only dental emergencies, with the use of personal protective equipment (PPE) for the treating dentists and dental assistants. For patients’ safety, it is of vital importance to take the proper history of the patients visiting the dental clinic regarding fever, cough, and sore throat.

In order to tackle the ongoing global calamity, lockdowns have been introduced as a measure to enhance social distancing. However, the public, not being used to such scenarios, have demonstrated anxiety and stress as well [9]. Since face-to-face contact poses a risk for the transmission of the coronavirus, telemedicine and teledentistry have been introduced, which deal with patients virtually when required [10]. Although standards have been set for the changes introduced in dental practice keeping in mind the current pandemic situation, dental practitioners have modified their practice according to their own expertise.

In this study, we aimed to evaluate patients’ satisfaction and the attitudes of dentists practicing in the midst of the COVID-19 pandemic, along with risk assessment.

## 2. Materials and Methods

The present study was approved by the ethical review committee of the Altamash Institute of Dental Medicine, Karachi. This study was conducted during April and July 2021, and previously approved by the Ethics Committee (protocol number: AID/ERC/03/2021/01). The Helsinki Declaration’s ethical aspects were followed. The study design was observational and comprised a convenient sample of dental patients and dental professionals. The Open-Epi calculator was used for sample size calculation. Considering the mean percentage of dental risk and fear at 78% [11], a confidence interval of 95% and margin of error of 5% were adopted in this study. The power of the test was 80. The estimated sample size calculated was 1422 participants.

The self-administered questionnaire was designed by the team of authors. The face, content, and construct validity of the questionnaire was assessed out by a team of researchers. The researcher team comprised a dental educationist and one member from each clinical dental specialty, i.e., an orthodontist member, a prosthodontist, periodontist, oral surgeon, and a restorative specialist. The reliability of the questionnaire was analyzed statistically through Cronbach’s alpha for the internal consistency of its items; for this purpose, 20% of the data were collected initially as a pilot study. The internal consistency of the questionnaire items according to Cronbach’s alpha revealed a strong correlation value of α = 0.851. The validated questionnaire was than disseminated electronically and in person among dental patients and dental professionals at public and private dental service centers in Karachi, Pakistan, between 2021 April and July 2021, through e-mail and other social media applications (WhatsApp^®^, Facebook^®^, Instagram^®^, Skype^®^, Imo messenger^®^, Snap Chat^®^).

**Figure 1:**
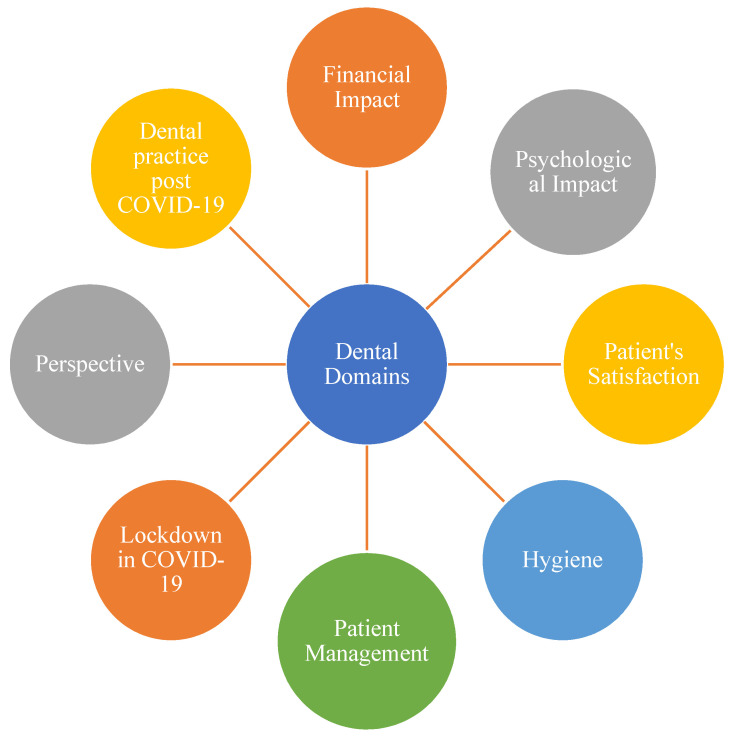
Diagram of dental domains.

The questionnaires consisted of two parts each related to the attitudes and practices amid the COVID-19 pandemic of the patients and dentists in the dental clinics. The first questionnaire covered patients’ demographic information and feedback, comprising a total of 15 closed questions related to the patient’s satisfaction towards COVID-19 security protocols when visiting the dental clinic; see Supplementary Materials (patients part), the detailed questionnaire utilized to collect reponses from patients in this study with relevant sections. The second questionnaire was based on the dental professional’s demographic information, and it contained a total of 45 items (Supplementary Materials (dentists part), the detailed questionnaire utilized to collect reponses from dentists in this study with relevant sections), which were classified under eight domains that encompassed the following: financial impact, psychological impact, patient satisfaction, hygiene, patient management, lockdown, dentist’s perspective while practicing dentistry in midst of pandemic, and post-COVID-19, as shown in Figure 1. The questions regarding dental domains consisted of financial impact, which considered how dental practice has been affected financially and the management of finances in the post-COVID-19 era. Secondly, questions regarding psychological impact focused on wearing PPE and fear of contracting the virus. Thirdly, patient satisfaction was assessed with questions regarding whether the patients were satisfied with the dental treatment or not and the environment of the dental practice. Fourthly, questions regarding hygiene focused on wearing PPE, oral hygiene instructions given to the patient, and the infection control protocol. Next, questions regarding patient management consisted of screening for fever, history of recent fever, and number of attendants per patient. Furthermore, questions regarding lockdown during the COVID-19 pandemic focused on the mental disturbance of patients during the pandemic and whether the dental practice should be closed during the pandemic situation. Next, questions regarding perspective focused on utilizing teledentistry, the use of rubber dams to minimize aerosol generation, changing face masks after every patient, and the use of aerosol suction. Lastly, questions regarding post-COVID-19 dental practice focused on changes in the dynamics of dentistry and how dentistry will evolve in the next few years. Regular reminders (at intervals of one week) were sent to the non-respondents via e-mail and other social media networks, after the initial distribution of the questionnaire.

SPSS-25 was used for statistical analysis. Descriptive statistics were performed for frequency, percentage, mean, and standard deviation of demographic variables, qualification of participants, experience, place of practice, and feedback from patients’ and dental professionals’ questionnaires. One-way ANOVA test was applied to determine the effect of the independent variables (age, gender, qualification, and years of experience) on the dependent variables (eight domains of the dental professional questionnaire). A multiple linear regression test was used to identify any significant relations of patients’ satisfaction with their demographic characteristics. A *p*-value of ≤0.05 was considered to be statistically significant.

## 3. Results

**Figure 2:**
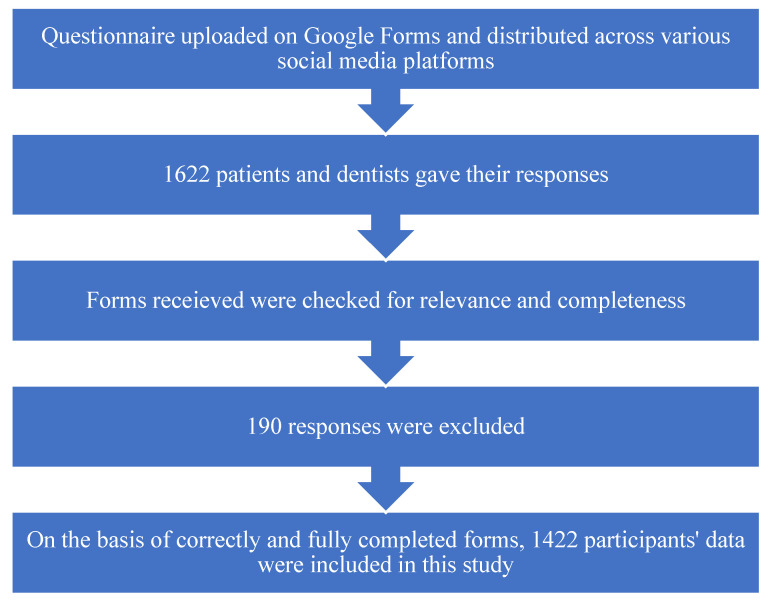
Flowchart of study participants’ recruitment.

In this observational study, we received a total of 800 forms from patients and 812 forms from dentists, among which 89 were excluded from the former and 101 from the later, based on irrelevance and being partially completed. The data of 711 patients and 711 dentists, a total of 1422 participants, were included in this study, as shown in Figure 2. Regarding the demographic characteristics of the patients, there were 380 (53.4%) males and 331 (46.6%) females, which belonged to the following age brackets: 197 were 25–35 years old, 134 were 35–45 years old, and 132 were 45–55 years of age. Half (50.5%) of the patients were educated, as presented in Table 1.

### 3.1. Patients’ Satisfaction

When visiting a dental clinic, the majority of the 584 (82.1%) patients agreed that clinic security staff were wearing a mask. Approximately 453 (63.47%) patients stated that they were provided with hand sanitization and 549 (77.2%) were thermally scanned to screen for fever. Most of the 483 (67.9%) patients felt comfortable in the dental clinic’s environment. Furthermore, 591 (83.1%) patients responded with “Yes” when asked about the implementation of social distancing in the clinic. Regarding the purpose of visit to the dental clinic, the majority of the 547 (76.9%) patients visited on the basis of a dental emergency. Regarding the patients’ concerns with the dentists, 605 (85.1%) patients reported dentists taking their medical history before starting treatment. Moreover, for protection, 553 (77.8%) patients responded with “Yes” when asked if the dentists were wearing PPE. Along with the dentist, more than half of the respondents (65.1%) saw dental assistants wearing PPE. However, the majority of the 564 (79.3) patients stated that they were not provided with PPE for protection. During treatment, many of the 487 (68.5%) patients saw no water droplets spreading in the air. After the treatment, nearly half of the patients (49.6%) stated that they were instructed by the dentist regarding hand hygiene. In the waiting area, 410 (57.7%) patients saw infection control measures to be taken, with 530 (74.5%) patients being satisfied with the services provided to them, as presented in Table 2.

For analysis of the relation of patients’ satisfaction with the independent variables (age, gender, and education), a multiple linear regression test was used. The outcome of the study showed that a weak correlation between patient satisfaction and independent variables existed. The regression model analysis for patient satisfaction (PS) and the independent variable fit showed a constant for R-Squared (R2) = 0.041 and adjusted R-Squared (AR2) was 0.0241; however, under the influence of external variables, only gender presented a significant difference. The patient satisfaction to gender beta (B) value was statistically significant (B = −0.203, *p* = 0.001), which indicates that gender had a 0.203 relationship with PS. Females were more concerned regarding the risk of contracting the coronavirus in the dental practice as compared to males in our study. On average, the effect of gender on PS was B_0_ = −0.005 in this study, as presented in Table 3.

Regarding the demographic characteristics of the dentists, there were 347 (48.8%) males and 364 (51.2%) females, which belonged to the following age brackets: 218 were 20–25 years old, 297 were 25–35 years old, and 102 were 35–45 years of age. The experience of the dentists was as follows: 0–5 years, 241 (33.9%); 5–10 years, 115 (16.2%); 10–15 years, 142 (20.0%). The qualification of the dentists was as follows: general dentist, 332 (46.7%), and specialist, 286 (40.2%), as presented in Table 4.

### 3.2. Financial Impact

When asked about financial planning for the long-term keeping in mind the COVID-19 pandemic, the majority of the 499 (70.2%) dentists agreed to plan their finances in a better way. Working with other dentists was stated by 422 (59.4%) dentists as a measure to distribute the fixed costs appropriately. To increase earnings, 416 (58.5%) dentists responded “Yes” to the question about investing more to attract more patients, and most of the 648 (91.1%) dentists further agreed to invest in ensuring patient safety.

### 3.3. Psychological Impact

Regarding psychological impact, 550 (77.4%) dentists experienced negativity, stress, an unhealthy diet, and disturbances in sleep patterns. Moreover, the majority of the 503 (70.7%) dentists experienced symptoms of post-traumatic stress disorder over the duration of the quarantine.

### 3.4. Patients’ Satisfaction

To keep the patients’ contact to a minimum, most of the 637 (89.6%) dentists agreed to inform the patients to avoid visiting the dental clinic if not urgent, with 677 (95.2%) dentists accepting one attendant per patient only. All of the dentists agreed to take a proper medical history and history of the symptoms of the patients with the inclusion of the COVID-19 safety checklist. Almost all of the 700 (98.5%) dental practitioners agreed with the implementation of lockdown if required.

### 3.5. Hygiene

Regarding maintaining proper hygiene, almost all of the dentists (98.3%) advised the use of electronic toothbrushes for immune-compromised patients. For protection against COVID-19, 659 (92.7%) dentists responded that they advised the patients regarding maintaining their personal hygiene. The majority of the 654 (92.0%) dentists agreed to give proper post-op instructions to the patients in order to avoid follow-up appointments, for the protection of the patients as well as the dentists. Before the start of the procedure, around 636 (89.5%) dentists recommended the use of 2% hydrogen peroxide mouthwash, along with 583 (82.0%) dentists ensuring regular cleaning of touched surfaces. During the treatment contact time with the patient, the majority of the 568 (79.9%) dentists used full PPE or a surgical gown, respirator, or face shield, with 565 (79.5%) dental practitioners advising the cleaning of floors with water and 1% sodium hypochlorite 5 min after a patient leaves. Lastly, the majority of the 660 (92.8%) respondents agreed to double wrap the disposables.

### 3.6. Patient Management

Most of the 580 (81.6%) dentists agreed that when entering the dental clinic, patients and those accompanying them should wear a face mask. Moreover, around 620 (87.2%) dentists asked their patients about the presence of fever or any associated symptoms consistent with COVID-19. Screening for fever was performed by 578 (81.3%) dental practitioners.

### 3.7. Lockdown in COVID-19 Pandemic

Most of the 654 (92.0%) dentists stated that their patients did suffer mentally due to the lockdown situation. To decrease the transmission of the virus, 523 (73.6%) dentists advised the closure of their clinics for everyone’s protection. For 641 (90.2%) dentists, this pandemic has been a learning experience.

### 3.8. Dentists’ Perspective

To decrease the spread of the virus, telehealth and teledentistry have been performed by 526 (74.0%) dental professionals. Although a respirator is required for protection, approximately 589 (82.8%) dentists felt that respirators decreased effective communication between the dentist and patient. In cases where intraoral X-rays are not required, the majority of the 594 (83.5%) dentists avoided it. When the tooth is no longer salvageable, most of the 600 (84.4%) dental practitioners agreed on their extraction. For the reduction of aerosols, approximately 653 (91.8%) dentists preferred the use of rubber dams, along with 539 (75.8%) dentists who used aerosol suction while using a high-speed handpiece. To minimize aerosol generation, many of the 596 (83.8%) dentists gave manual scaling as a preference over ultrasonic scaling. Furthermore, 632 (88.9%) dentists preferred changing their masks after every other patient. To improve ventilation, 483 (67.9%) dentists used air conditioners with open windows. To lessen the cost burden of protection of the patients, 581 (81.7%) dentists agreed that these costs should be covered by the patients’ insurance companies. Moreover, slightly over half of the dentists suggested the advantage of using mobile dentistry.

### 3.9. Practicing Dentistry Post-COVID-19

Keeping in mind the dental practice before the beginning of the COVID-19 pandemic, the majority of the 554 (77.9%) dentists stated that the key dynamics of dentistry will change after COVID-19. Furthermore, approximately 362 (50.9%) dentists stated that they had noted changes in their profession and profits in their practices. Lastly, 453 (63.7%) expressed that they anticipated a change in dental practice in the next 5 years.

For the possible relation of age, gender, experience, and qualification with eight dental domains, a one-way ANOVA test was used. Regarding gender, a significant relation was noted in all of the dental domains (*p*-value ≤ 0.001) except post-COVID-19, as presented in Table 5. Regarding age, a significant relation was noted in all of the dental domains (*p*-value ≤ 0.001) except perspective, as presented in Table 6. For qualification, a significant relation was noted in all dental domains (*p*-value ≤ 0.001) except patient management, as presented in Table 7. Lastly, for experience, a significant relation was noted in all of the dental domains (*p*-value ≤ 0.001), as presented in Table 8.

## 4. Discussion

At the present moment, coronavirus is spreading globally, with vaccination programs being carried out to tackle it. Many aspects of human life have been affected, with the dental practice being one of them. Since dentists, as well as patients, are directly exposed to the possible risk of contracting the virus, many risk factors, as well as aspects, have changed in dental practice.

To protect the patients when visiting dental clinics, many precautionary measures have been implemented, such as the use of masks, temperature screening, and hand hygiene at the clinic. Individuals themselves are knowledgeable regarding the protective measures to be taken, and similar findings are present in the literature [12]. Many of the patients in this study were satisfied with the precautionary measures implemented for their safety. However, these results contrast with those of a study in the literature where the majority of the patients were concerned about infection control [13]. In the literature, the 3Ps model has been used, which is Preparation, Protection, and Prevention, as a systemic approach in order to protect healthcare staff as well as patients [14].

According to the latest guidelines issued by the CDC, dental practices have been modified according to the current coronavirus situation in different countries [8]. Patients in this study mostly only visited dentists when they had a dental emergency. These results correspond with a study in the literature with similar findings, mainly due to fear of contracting the virus [15]. The anxiety of the patients visiting the dental clinics primarily depends upon the use of precautionary measures being followed by the dental staff. In this study, the majority of the patients reported seeing the dentists and their staff wearing PPE, although PPE was not provided for the patients. Similar findings have been reported in a study by Dosup Kim et al., where healthcare professionals wore complete PPE [16].

Financially, dentists have been affected mainly due to the increased costs of implementing protective measures against COVID-19. Dentists in this study suggested better planning of their finances for the long term, along with the need to invest more in order to ensure the better safety of the patient. A study in the literature found that dental professionals are suffering financially from the increased demand of treatment due to the extra protective measures being taken [17].

It is widely known that the coronavirus has caused a great array of psychological effects, not only on the general population but on dental professionals as well [9]. Dentists in our study suffered a great amount of stress and disturbance in sleep patterns while working during the current pandemic situation, with some experiencing post-traumatic stress disorder symptoms as well. Similar results have been found in a study where dental professionals suffered psychologically, regardless of whether they worked with patients or not [18]. This could be due to concerns regarding being affected by the virus, along with the unintentional spread of the virus to other individuals in close proximity.

Patient satisfaction has been vital in treating patients in the midst of the COVID-19 pandemic, mainly due to coronaphobia, and dental clinics pose a high risk of contracting the virus. Patients with dental emergencies visit the dental clinic with severe pain, so almost all the dentists in our study were willing to treat such emergencies during the lockdown situation. Furthermore, to control the transmission of the virus, the majority of the dentists allowed one attendant per patient. Such findings correspond with a study by Bruna et al., where dentists mostly only carried out dental emergency procedures [19]. Proper history taking was made compulsory by all of the dentists in this study before starting any dental procedures. Such conclusions correspond to a study in Saudi Arabia, where the dental practitioners were vigilant while taking the history of their patients [20].

In order to decrease patients’ exposure and time in the dental clinics, it was concluded from our study that the dentists gave proper post-op instructions to the patients to avoid a follow-up visit when not required. Moreover, proper cleaning of surfaces with water and 1% sodium hypochlorite after every patient was implemented by the practicing dentists. Such similar practices have been followed by dentists in order to prevent the transmission of the coronavirus [21].

Taking patients and their attendants’ temperature and ensuring the wearing of face masks, along with taking the history of fever, have become the new norm among dentists in their clinics. This is due to the majority of the dentists themselves being afraid of contracting the virus while treating patients [22]. To protect themselves as well as the patients, the majority of the dentists in our study opted for the use of teledentistry when required in non-emergency cases. Similarly, patients are also afraid to contract the virus, so they also welcome the use of teledentistry [23]. Such findings correspond to one study in the literature where teledentistry was successfully used by the dentists and they encouraged its use in their dental practice [24].

It is well established that most dental procedures generate aerosols, which are the primary modality of transmission of the coronavirus. To counteract this, many dentists in our study used aerosol suction, preferred the use of manual scaling instead of ultrasonic scaling, and changed their masks after every patient. Such conclusions have been well stated in the literature in studies that also recommend minimal generation of aerosols whenever possible [25]. Moreover, one study reported that a high risk of contracting the coronavirus does exist among highly aerosol-generating dental subspecialties such as periodontics [26].

Keeping in mind the current situation, the dynamics of performing dentistry have changed significantly, as suggested by the dental professionals in this study, with further changes expected in the next 5 years. Fear and stress have been generated to a greater extent, which previously were absent amongst dentists in the pre-COVID-19 era [27].

Despite the strengths of this study, such as the inclusion of a large sample of dental practitioners and patients, this study was met with some limitations. Firstly, since convenience sampling was used, there is a potential of bias. Lastly, since the data were collected from the participants in a short period of time, the attitudes and practices of patients and dentists may have altered with further research being carried out on COVID-19.

## 5. Conclusions

At the present moment, both dental professionals as well as patients are very familiar with the precautionary measures necessary to reduce COVID-19 transmission. Furthermore, changes in operational dynamics along with risk assessment have been introduced in dental practice for the better management and safety of the healthcare professionals and the patients. Proper management of the risk factors has proven to be the primary modality by which the risk of contracting the virus can be decreased in dental clinics.

## Figures and Tables

**Table 1:** Demographic characteristics of the patients (n = 711).

	Variables	N	%
Age	10–25 25–35 35–45 45–55 55–65 65–75	80 197 134 132 95 73	11.3 27.7 18.8 18.6 13.4 10.3
Gender	Male Female	380 331	53.4 46.6
Education	Educated Non-educated	359 352	50.5 49.5

**Table 2:** Responses of the patients to assess their satisfaction.

Questions	Responses	
	Yes	No
Was the clinic’s security wearing mask?	584	127
Did you receive preventive measures at the clinic’s entrance?	453	258
Was proper social distancing practiced at the clinic?	591	120
Did you arrive at the clinic with Dental emergency?	547	164
Was PPE provided to you for your safety?	147	564
Was the dental assistant wearing proper PPE?	463	248
Was the dentist wearing proper PPE?	553	158
Was proper medical history taken?	605	106
Were infection control measures performed in the waiting area?	410	301
Are you satisfied with the services provided to you?	530	181

**Table 3:** Comparison of patient’s satisfaction (PS) with demographic characteristics (n = 711).

Variables	Unstandardized Coefficients		Standardized Coefficients	t	*p*-Value	95.0% Confidence Interval for B		Collinearity Statistics	
	B	Std. Error	Beta			Lower Bound	Upper Bound	Tolerance	VIF
Age	−0.005	0.005	−0.039	−1.064	0.288	−0.014	0.004	0.997	1.003
Gender	−0.076	0.014	−0.203	−5.506	** 0.001	−0.104	−0.049	0.994	1.006
Education	0.010	0.014	0.026	0.718	0.473	−0.017	0.037	0.997	1.003

B = The rate of change per unit between dependent and independent variables: patient’s satisfaction, age, gender, and education; VIF = variance inflation factor, denotes the amount of multicollinearity in model; t = test of the regression coefficients; ** *p*-value < 0.001; B_0_ = unstandardized coefficient, i.e., average estimation of age, gender, and education with patient satisfaction. For PS, the constant for R-Squared (R2) = 0.041 and Adjusted R-Squared (AR2) was 0.0241.

**Table 4:** Demographic characteristics of the dentists (n = 711).

	Variables	N	%
Age	20–25 25–35 35–45 45–55 55–65	218 297 102 32 62	30.7 41.8 14.3 4.5 8.7
Gender	Male Female	347 364	48.8 51.2
Experience	0–5 years 5–10 years 10–15 years 15–20 years 20–25 years 25–30 years Above 30 years	241 115 142 88 56 63 3	33.9 16.2 20.0 12.4 7.9 8.9 0.8
Qualification	General Dentist Specialist Consultant	332 286 93	46.7 40.2 13.1

**Table 5:** Comparison of gender with dental domains.

Variables	Df	Mean Square	*p*-Value
Financial Impact	1	6.903	0.001
Psychological Impact	1	6.357	0.001
Patient’s Satisfaction	1	0.611	0.001
Hygiene	1	0.434	0.001
Patient Management	1	0.245	0.013
Lockdown	1	1.038	0.001
Perspective	1	0.411	0.010
Post-COVID-19	1	0.039	0.564

Df: difference or degree of freedom between gender and dental domains in this study; *p*-value ≤ 0.05 was considered significant.

**Table 6:** Comparison of age with dental domains.

Variables	Df	Mean Square	*p*-Value
Financial Impact	4	9.939	0.001
Psychological Impact	4	1.875	0.001
Patient’s Satisfaction	4	0.086	0.001
Hygiene	4	0.639	0.001
Patient Management	4	0.926	0.001
Lockdown	4	0.342	0.001
Perspective	4	0.099	0.168
Post-COVID-19	4	3.560	0.001

Df: difference; *p*-value ≤ 0.05 was considered significant.

**Table 7:** Comparison of qualifications with dental domains.

Variables	Df	Mean Square	*p*-Value
Financial Impact	2	14.568	0.001
Psychological Impact	2	4.239	0.001
Patient’s Satisfaction	2	0.142	0.001
Hygiene	2	0.273	0.001
Patient Management	2	0.101	0.077
Lockdown	2	1.231	0.001
Perspective	2	0.376	0.002
Post-COVID-19	2	2.940	0.001

Df: difference; *p*-value ≤ 0.05 was considered significant.

**Table 8:** Comparison of experience with dental domains.

Variables	Df	Mean Square	*p*-Value
Financial Impact	6	5.038	0.001
Psychological Impact	6	2.360	0.001
Patient’s Satisfaction	6	0.035	0.001
Hygiene	6	0.031	0.028
Patient Management	6	0.211	0.001
Lockdown	6	0.907	0.001
Perspective	6	0.378	0.001
Post-COVID-19	6	0.473	0.001

Df: difference; *p*-value ≤ 0.05 was considered significant.

## Data Availability

The data included in the present study are available upon request from the corresponding author.

## Supplementary Materials

The following are available online at https://www.mdpi.com/article/10.3390/ijerph182212244/s1, Operational Implications and Risk Assessment of COVID-19 in Dental Practices (patients part), Operational Implications and Risk Assessment of COVID-19 in Dental Practices (dentists part).

## Abbreviations

Abbreviation	Full Name/Description
CDC	Centre for Disease Control and Prevention
PPE	Personal Protective Equipment
COVID-19	Novel Coronavirus Disease 2019
