# Supplementary material for: Operational Implications and Risk Assessment of COVID-19 in Dental Practices
Source: Int J Environ Res Public Health. 2021 Nov 22;18(22):12244. doi: 10.3390/ijerph182212244 (PMC8619992; doi:10.3390/ijerph182212244)
Supplement: Supplementary file 1 [file ijerph-18-12244-s001.zip › Appendix A (Patients part).pdf]

# Operational Implications and Risk Assessment of COVID-19 in Dental Practices

We invite you to fill this survey questionnaire for our study and thank you in advance for your participation.

Please indicate your consent: I have read the invitation letter and I have understood the nature of this proposed study. I Consent to participate in the study. I understand that my participation in this study will not lead to any financial benefits.

- ○ Yes
- ○ No
- ○ Other

| | |
|---|---|
| **Patients Bio-data** | |
| **Age** | |
| **Gender** | |
| **Occupation** | |
| **Patient M.R Number (if applicable)** | |

## Dear patients,

We need your feedback on what we can do to serve you better

| Questionnaire items | Yes | No |
|---|---|---|
| Did any restrictions placed by clinic's security regarding to wear a face mask and to limit the number of attendants? | | |
| Did you receive any preventive measures at clinic entrance e.g., hang hygiene? | | |
| Upon arrival, did you feel comfortable with the environment? | | |
| Were you seated accordingly with proper distancing? | | |
| Did it take long to wait for your appointment? | | |
| Did you have any dental emergency? | | |
| Was thermal scanning done before you entered the clinic? | | |
| Did the dentist take any medical history before starting the procedure? | | |
| When you entered the procedure room was the dentist wearing proper personal protective hygiene (PPE) | | |
| Was the dental assistant wearing proper PPE as well? | | |
| Were any PPE provided for your safety? | | |

| | | |
|---|---|---|
| How was the aerosol procedure performed, were the water droplets spreading in the air? | | |
| Did the dentist guide the importance of proper hand hygiene? | | |
| Were proper infection control measures followed in the waiting area? | | |
| Are you satisfied with the services being provided to you? | | |

**Thank you.**
