# Supplementary material for: Operational Implications and Risk Assessment of COVID-19 in Dental Practices
Source: Int J Environ Res Public Health. 2021 Nov 22;18(22):12244. doi: 10.3390/ijerph182212244 (PMC8619992; doi:10.3390/ijerph182212244)
Supplement: Supplementary file 1 [file ijerph-18-12244-s001.zip › Appendix B (Dentists part).pdf]

Operational Implications and Risk Assessment of COVID-19 in Dental Practices

We invite you to fill this survey questionnaire for our study and thank you in advance for your participation.

Please indicate your consent: I have read the invitation letter and I have understood the nature of this proposed study. I Consent to participate in the study. I understand that my participation in this study will not lead to any financial benefits.

- ○ Yes
- ○ No
- ○ Other

Demographic data

**Gender**			
**Age**			
**Experience**			
**Qualification**	GD	Specialist	Consultant

	**Yes**	**No**
**Public sector practitioner**		
**Private section practitioner**		

**Works in:**	Private clinic	Hospital

Financial impact

	**Yes**	**No**
**After COVID-19 do you think we need to plan our finances better for the long term?**		
**Do you think working along with the associates will help distribute the fixed costs more appropriately?**		
**Do we need to invest more to attract patients**?		
**Do we need to invest further so that the patient feels safer?**		

Psychological impact

	Yes	No
**Do negativity, stress, unhealthy diet & sleep pattern effect disease pattern**		
**Symptoms of post-traumatic stress disorder (PSTD) have been positively associated with the duration of quarantine**		

Patient's Satisfaction

	Yes	No
**Should all patients with pre-booked appointments be contacted and advised to avoid attending if non-urgent cases.**		
**Will you accept urgent cases during lockdown?**		
**Do you agree that a patient should be accompanied by only one person**		
**Do you agree that a patient should be accompanied by only one person**		
**When compiling patient notes, should history of the symptoms be noted**		
**Should a additional section be included for the COVID-19 safety checklist?**		

Hygiene

	Yes	No
**Would you advise immune-compromised patients to use electronic toothbrush?**		
**Should patients be provided with advice regarding ongoing personal hygiene as a preventative measure?**		
**Patient provided with appropriate after-care instructions to avoid follow-up appointments where possible**		
**Should non-disposable toothbrush be cleaned with anti-microbial agent like Chlorhexidine**		
**Should a 2% hydrogen peroxide mouthwash be used prior to procedure**		
**Regular cleaning of touched surfaces**		
**Do mouth wash or dental floss help in the prevention of virus spread?**		
**Use of full PPE or surgical gown, respirator, face shield**		
**Cleaning of floors 5 minutes after patient leave with water and 1% sodium hypochlorite**		
**Disposables should be double wrapped**		

Patient Management

	Yes	No
**When entering the hospital, patients and anyone accompanying them should wear a face mask.**		
**Ask about the presence of fever or other symptoms consistent with COVID-19**		
**Take the patient's temperature**		
**Triage all patients in need of dental care to reduce patients wait times in the waiting area.**		

Lockdown in COVID-19 pandemic

	Yes	No
**Are your patients mentally effected after lock down**		
**Have you practiced or learnt anything after the pandemic**		
**Do you agree of dental clinics closure during lockdown**		

Your Perspective

	Yes	No
**Telehealth / Telemedicine**		
**Change in mindset for professional and scientific approach.**		
**Do you think wearing a respirator causes hindrance in effective communication between you and the patient?**		
**Avoid intra oral X-ray**		
**A tooth is extensively carious go for extraction**		
**Rubber dam (non-latex) for RCT**		
**Aerosol suction while using high speed hand piece**		
**Manual scaling verses ultrasonic scaling**		
**Change of face mask after every patient**		
**Use of air conditioner along with open windows for ventilation**		
**Use of anti-retraction hand piece**		
**Do we pass the cost of increased spending on safety equipment e.g., PPEs to the patients / patient's insurance companies?**		
**Is mobile dentistry a competitive advantage**		

Practicing Dentistry Post-COVID-19

	Yes	No
**Is mobile dentistry a competitive advantage**		
**Will there be changes in professional and profitable practice.**		
**Will there be a change of scenario over the next 5 years**		

Thank you.
